# High Lithium Ion Transport Through rGO-Wrapped LiNi_0.6_Co_0.2_Mn_0.2_O_2_ Cathode Material for High-Rate Capable Lithium Ion Batteries

**DOI:** 10.3389/fchem.2019.00361

**Published:** 2019-05-28

**Authors:** Wook Ahn, Min-Ho Seo, Tuan Kiet Pham, Quoc Hung Nguyen, Van Tung Luu, Younghyun Cho, Young-Woo Lee, Namchul Cho, Soon-Ki Jeong

**Affiliations:** ^1^Department of Energy Systems Engineering, Soonchunhyang University, Asan-si, South Korea; ^2^New and Renewable Energy Research Division, Hydrogen and Fuel Cell Center, Korea Institute of Energy Research, Daejeon, South Korea

**Keywords:** lithium ion battery, graphene-based cathode composite, nickel-rich, LiNi_0.6_Co_0.2_Mn_0.2_O_2_, galvanostatic intermittent titration technique

## Abstract

In this work, we show an effective ultrasonication-assisted self-assembly method under surfactant solution for a high-rate capable rGO-wrapped LiNi_0.6_Co_0.2_Mn_0.2_O_2_ (Ni-rich cathode material) composite. Ultrasonication indicates the pulverization of the aggregated bulk material into primary nanoparticles, which is effectively beneficial for synthesizing a homogeneous wrapped composite with rGO. The cathode composite demonstrates a high initial capacity of 196.5 mAh/g and a stable capacity retention of 83% after 100 cycles at a current density of 20 mA/g. The high-rate capability shows 195 and 140 mAh/g at a current density of 50 and 500 mA/g, respectively. The high-rate capable performance is attributed to the rapid lithium ion diffusivity, which is confirmed by calculating the transformation kinetics of the lithium ion by galvanostatic intermittent titration technique (GITT) measurement. The lithium ion diffusion rate (*D*_Li_) of the rGO-wrapped LiNi_0.6_Co_0.2_Mn_0.2_O_2_ composite is *ca*. 20 times higher than that of lithium metal plating on anode during the charge procedure, and this is demonstrated by the high interconnection of LiNi_0.6_Co_0.2_Mn_0.2_O_2_ and conductive rGO sheets in the composite. The unique transformation kinetics of the cathode composite presented in this study is an unprecedented verification example of a high-rate capable Ni-rich cathode material wrapped by highly conductive rGO sheets.

## Introduction

With the increasing environmental concerns such as energy depletion and gas emission problems, interest in novel energy storage systems (ESSs) and renewable energy such as photovoltaic and wind power is at an all-time high (Li et al., 2015; Zhao et al., 2015). As the world population continues to grow, we could not fulfill the energy consumption requirement without developing a clean energy system (Majeau-Bettez et al., 2011; Catenacci et al., 2013). To address this, many research groups have recently been contributing to the development of electrochemical energy conversion and storage devices such as hybrid capacitors, metal–air batteries, and high-power lithium ion batteries (Lim et al., 2015; Ahn et al., 2016a,b, 2018; Seo et al., 2018). Among them, technology advancement in high-power lithium ion batteries that can be applied to electric vehicles (EVs) has been investigated (Lin et al., 2017; Cano et al., 2018; Fu et al., 2018). LiCoO_2_ and LiNi_1/3_Co_1/3_Mn_1/3_O_2_ have been used as conventional cathode materials for mobile devices because of their stable cyclability with ease of manufacture (Venkateswara Rao et al., 2011; Byeon et al., 2018). However, there are still issues on whether conventional cathode materials can improve energy and power density for ESSs and EVs. A strong candidate for a high-energy and high-power-density material is the Ni-rich LiNi_0.6_Co_0.2_Mn_0.2_O_2_ layered material, which has a practical capacity of *ca*. ~190 mAh/g (Kim et al., 2015; Shim et al., 2017; Fu et al., 2018; Liao et al., 2018). Also, this Ni-rich material has the advantages of being relatively cheap and environmental friendly. However, this material still has the critical problem of having low electrical conductivity with poor cycle life, which is difficult to apply to ESSs and EVs for high power density with long cycle stability. To overcome such challenges, numerous research have been focusing on the development of element-doped materials and carbon-based (carbon coating, mixing, etc.) composites (Ju et al., 2014, 2018; Lim et al., 2014a, 2015).

Particularly, a graphene (rGO)-based cathode composite is prepared by various chemical reaction routes to demonstrate high-rate capability for lithium ion batteries. Most reported studies on high-power composites have been limited to aggregated mixture of cathode either dispersed in the graphene matrix or wrapped by graphene sheets (Yang et al., 2012; Kucinskis et al., 2013; Lim et al., 2014b; Shim et al., 2017). Furthermore, an in-depth study with emphasis on confirming the transformation kinetics of lithium ion during charge–discharge to further investigate the rate capability tendency of cathode material has never been reported.

Here, we introduce an ultrasonication-assisted self-assembly route under a surfactant solution environment, where the final morphology of rGO-wrapped LiNi_0.6_Co_0.2_Mn_0.2_O_2_ (NCM622) forms a homogeneous nanoparticle interconnected with a thin layer of rGO nanosheets. Furthermore, practical transformation kinetics of LiNi_0.6_Co_0.2_Mn_0.2_O_2_ during lithium ion intercalation–deintercalation advances will be verified by galvanostatic intermittent titration technique (GITT) measurement.

## Experimental

### Synthesis of the rGO-Wrapped LiNi_0.6_Co_0.2_Mn_0.2_O_2_ Composite

The detailed synthesis procedure for LiNi_0.6_Co_0.2_Mn_0.2_O_2_ preparation was followed by our previous research paper using a combustion synthesis method (Ahn et al., 2014a). The appropriate NH_2_CONH_2_ (urea):nitrate ratio (3:2 mol/mol) dissolved in deionized (DI) water is effective for preparing nanostructured material, and it confirmed that high crystalline LiNi_0.6_Co_0.2_Mn_0.2_O_2_ was synthesized at the sintering temperature of 800°C on that study. The rGO was also synthesized based on the previous methodology—called the modified improved hummers' method—where the rGO consists of under 4.2 layers of graphene sheets, resulting in high electrical conductivity and surface area (Ahn et al., 2014b, 2016a). For the preparation of rGO-wrapped LiNi_0.6_Co_0.2_Mn_0.2_O_2_, 0.1 g of rGO powder was firstly dispersed into 150 ml of DI water and then 2 ml of 1% Triton X-100 surfactant was added into the solution with vigorous stirring for 30 min to functionalize the hydrophilic nature of the rGO surface. In another beaker, 0.9 g of pristine NCM622 (LiNi_0.6_Co_0.2_Mn_0.2_O_2_) active material was dispersed into 150 ml of DI water, and ultrasonication was carried out to pulverize the aggregated bulk powder for the preparation of primary nanoparticles at an energy of 100 kJ. Then, the NCM622 solution was added into the activated rGO dispersed solution and sonicated for 1 h to prepare a homogenously self-assembled composite.

## Characterizations

To confirm the structure and crystallinity of each material, X-ray diffraction (AXS D8 Advance, Bruker) of the phases was carried out with Cu Kα radiation (λ = 1.5405 Å) in the 2θ range of 5–80° with 0.02° intervals, at a 2° min^−1^ scanning rate. The morphologies and microstructures were analyzed using a scanning electron microscope (SEM; S400, Hitachi), and high-resolution transmission electron microscopy (HR-TEM; JEOL 2010F, JEOL Ltd.) was carried out to confirm the microscopic images of synthesized materials. To ascertain the binding energy and verify the oxidation states of each transition metal element, X-ray photoelectron spectroscopy (XPS) was conducted (K-Alpha XPS spectrometer, Thermal Scientific). For electrochemical testing, the cathode electrodes were prepared by mixing 10 wt.% of poly(vinylidene fluoride-co-hexafluoropropylene) (PVDF-co-HFP, KYNAR® 2801) binder, 5 wt.% of Super-P (C65 super-P, Timcal co. LTD) conductive carbon, and 85 wt.% of the rGO-wrapped NCM622 composite in *N*-methyl-2-pyrrolidone (NMP); then, this slurry was mixed and coated on Al foil (20 μm) to a 60-μm thickness of mixed slurry using a doctor blade to coat a uniform cathode electrode. The electrode was dried in oven at 60°C for 24 h and then pressed using a twin roller. The final thickness of the cathode material on the Al foil was 45 μm. CR2032 coin-type cells were assembled using 1.0 M solution of LiPF_6_ dissolved in a mixture of ethylene carbonate and diethyl carbonate (EC/DEC, 50:50 vol.%) as an electrolyte and Celgard 2400 as a separator. A lithium chip was used as the counter electrode for the half-cell evaluation. The electrode size is 14Ø (electrode loading mass: 2.20 mg/cm^2^, loaded mass based on active material: 1.76 mg/cm^2^) and 50 μl/mg of the electrolyte was injected into the coin cell. The coin cell assembly procedure was completely performed in an argon (Ar)-filled glove box. The electrochemical evaluation was carried out (CT2001C, LANHE, China) at a current density of 20 to 1,000 mA/g with a voltage range between 3.0 and 4.3 V at room temperature. GITT of the coin cell with NCM/rGO material was also carried out to investigate the evolution of lithium diffusivity as a function of induced potential at the state of charge and discharge with a battery tester at room temperature.

## Results and Discussion

**Figure 1:**
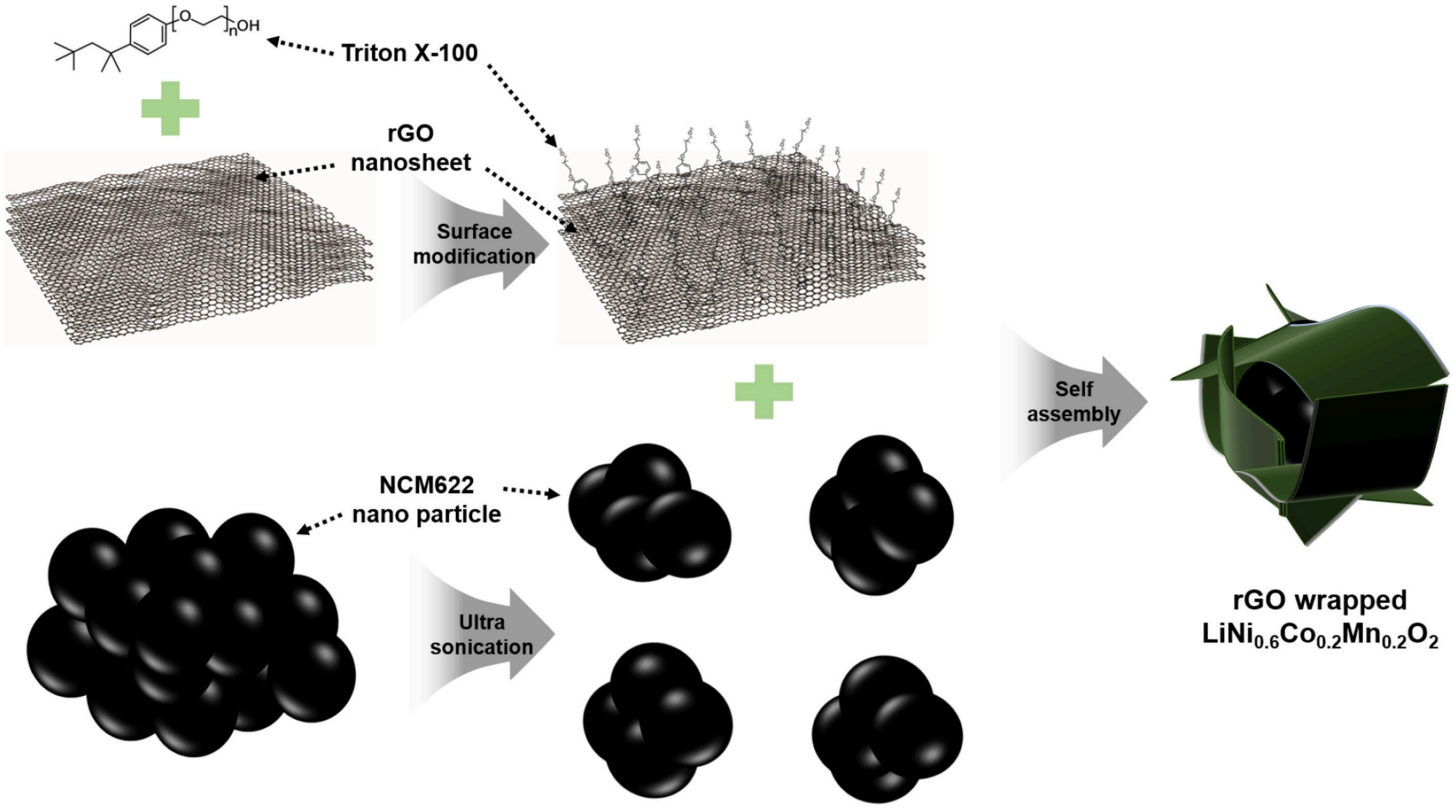
The schematic synthesis procedure of the rGO-wrapped NCM622 composite. The unique NCM622/rGO composite is prepared by a facile self-assembly method using a Triton X-100 surfactant. The hydrophilic part of the rGO sheet easily connects to the NCM622 nanoparticle, effectively decreasing overall contact resistance.

The schematic illustration of a facile synthesis procedure for the NCM/rGO composite is presented in Figure 1. The rGO nanosheet of approximately under 4.2 layers is functionalized with Triton X-100 surfactant to create hydrophilic surface on the rGO, after which, agglomerated rGO powder is homogeneously dispersed into DI water. Triton X-100 is a well-known surfactant that has hydrophilic and hydrophobic parts at both ends of the chain, respectively, resulting in an increase in hydrophilicity of rGO sheets, and this surfactant could help obtain the rGO-wrapped composite (Patey et al., 2009). For the preparation of NCM622, we could effectively synthesize the secondary bulk material which consists of primary nanoparticles from the combustion synthesis method. In order to materialize a high mass loading electrode, a relatively bulk micron-sized particle is better than a nanosized particle; however, the aggregated powder is unfavorable for preparing surface modification such as an rGO-wrapped or a carbon-based material coated composite. Therefore, ultrasonication is used to pulverize the bulk secondary particle of NCM622. On the other hand, ultrasonication treatment affects the morphological change of a wrinkled rGO sheet, resulting in a flattened rGO sheet, which has more surface exposure to the NCM-rich environment in the solution (Ahn et al., 2014b). Finally, the ultrasonication-assisted self-assembly methodology successfully obtains a homogeneously rGO-wrapped NCM composite.

**Figure 2:**
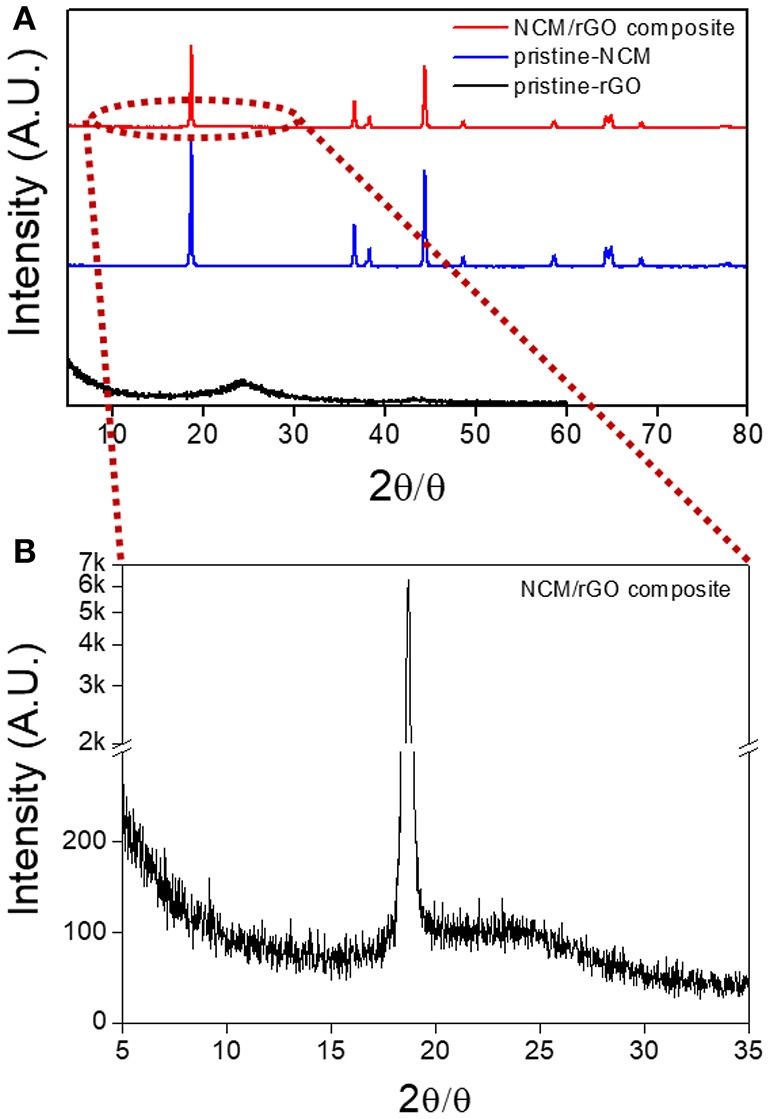
XRD patterns of **(A)** NCM/rGO composite, pristine NCM, and pristine rGO materials; **(B)** the inset image of the NCM/rGO composite in the range of 5–35° to confirm the specific rGO peak. The XRD patterns of the NCM/rGO composite show peaks that closely match those of pristine NCM and pristine rGO without any impurity peaks.

The XRD patterns obtained with the NCM/rGO composite, pristine NCM, and pristine rGO are presented in Figure 2A, and the result of the NCM/rGO composite is consistent with results from the mixed XRD patterns of pristine NCM and pristine rGO. All the characteristic peaks of the NCM/rGO composite show a hexagonal structure with an R-3m space group (Ahn et al., 2014a; Salitra et al., 2018). From the inset image (Figure 2B) of XRD patterns in the range of 5–35°, the characteristic broad diffraction peak of rGO indicates that rGO is homogeneously mixed with the NCM material, resulting in strong ultrasonication treatment that did not generate the phase transformation or decomposition of the NCM material during the self-assembly procedure. The lattice constant (*a, c*) of NCM/rGO was calculated following Bragg's law, with values of 2.895 and 14.287 Å, respectively. The high *c/a* (4.939) ratio is related to the well-defined hexagonal layered structure, and *c* represents the distance of a metal–metal interslab, which is directly associated with crystallinity, verifying that the NCM/rGO nanocomposite obtains a high crystalline structure (Ahn et al., 2014a). In order to estimate the cation mixing of the sample, the *I*_(003)_*/I*_(104)_ peak ratio was calculated, and NCM/rGO from this study shows 1.35, which supports the well-crystalline structure without cation mixing.

**Figure 3:**
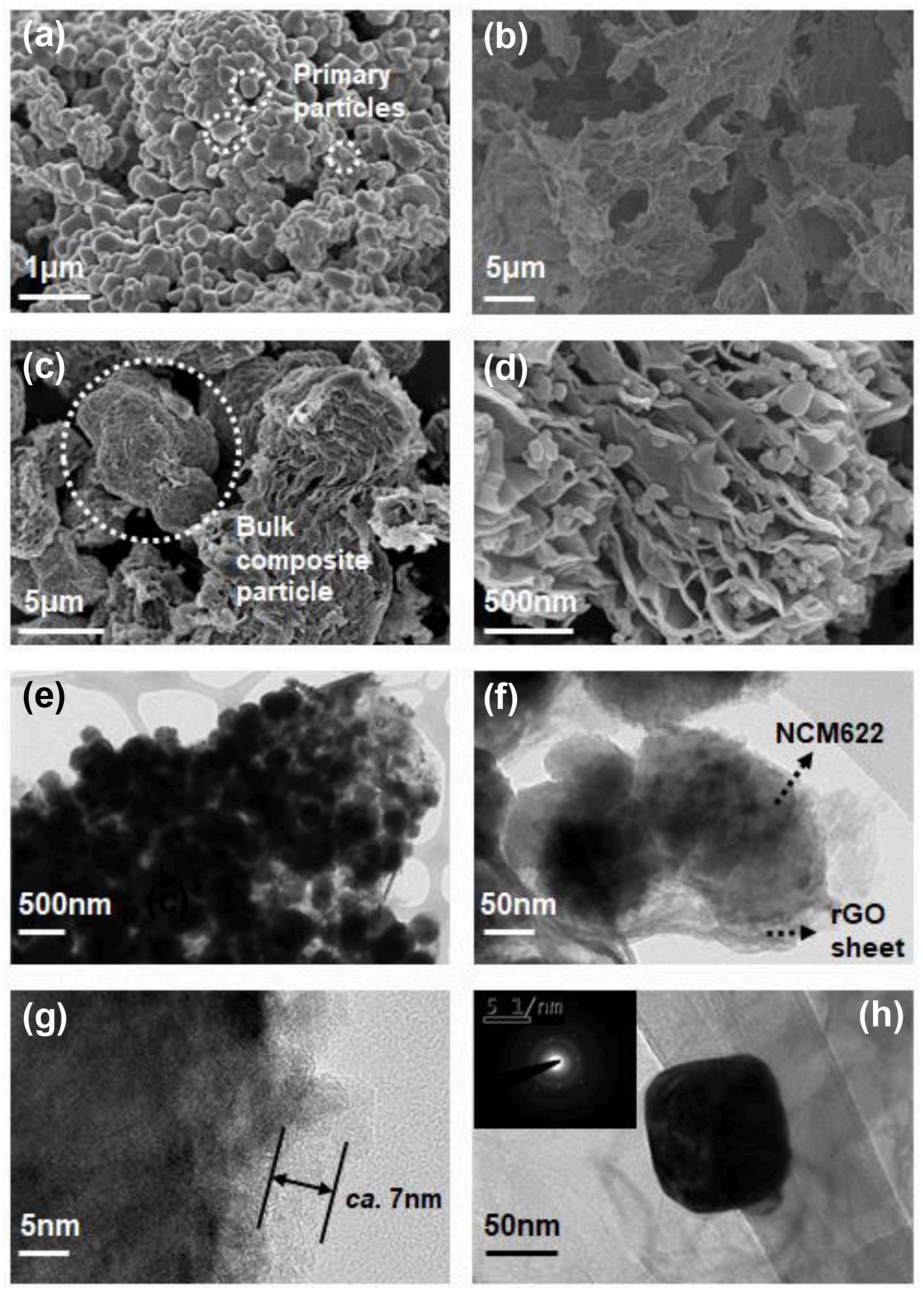
SEM images of various morphologies of **(a)** pristine NCM, **(b)** rGO, and **(c)** NCM/rGO without ultrasonication procedure, and **(d)** NCM/rGO composite ultrasonication-assisted self-assembly. The microscopic morphologies of the NCM/rGO composite **(e–h)**; the NCM/rGO composite without ultrasonication forms aggregated bulk particles after the self-assembly procedure.

Morphological analyses with SEM and TEM images are presented in Figure 3. Figure 3a shows the bulk secondary particle by forming the aggregated morphology of the primary particle for the NCM material with a dense structure, facilitating a high mass loading electrode. The bulk secondary particle, however, has a disadvantage in surface modification, and the rGO nanosheet could not virtually cover the entire bulk particle. Therefore, ultrasonication is essentially required to pulverize the bulk secondary particle into a nanosized primary particle to be modified further. Figure 3b shows pristine rGO nanosheets with numerous wrinkles; the crumpled structure is derived from the high surface energy, indicating an agglomerated structure throughout the rGO sheets. The NCM/rGO composites without and with ultrasonication assistance are presented in Figures 3c,d respectively. From the result, aggregated bulk NCM/rGO cannot be homogeneously dispersed into the solution, and then the final product exists mostly in a separated phase. It is verified that the ultrasonication-assisted procedure effectively contributes to the preparation of the homogeneous rGO-wrapped NCM composite, and TEM images support this result. TEM analysis of the NCM/rGO composite under ultrasonication-assisted self-assembly has been conducted (Figures 3d–h), where the average particle size of the NCM/rGO composite is observed to be ~100–150 nm, as shown in Figures 3e,f. The wrapped rGO sheet observed in a high-resolution TEM image (Figure 3g) shows a clear indication that the rGO sheet has been successfully covered throughout the NCM particle with ~7 nm of rGO thickness. Further diffraction analysis (Figure 3h) confirms that the SAED pattern of NCM has no phase transition after the strong ultrasonication-assisted self-assembly process, whose SAED pattern of NCM is obviously in accordance with our previous result (Ahn et al., 2014a). Finally, the resultant NCM/rGO composite with ultrasonication process effectively obtains a homogeneous rGO-wrapped NCM cathode material without damaging NCM primary nanoparticles.

**Figure 4:**
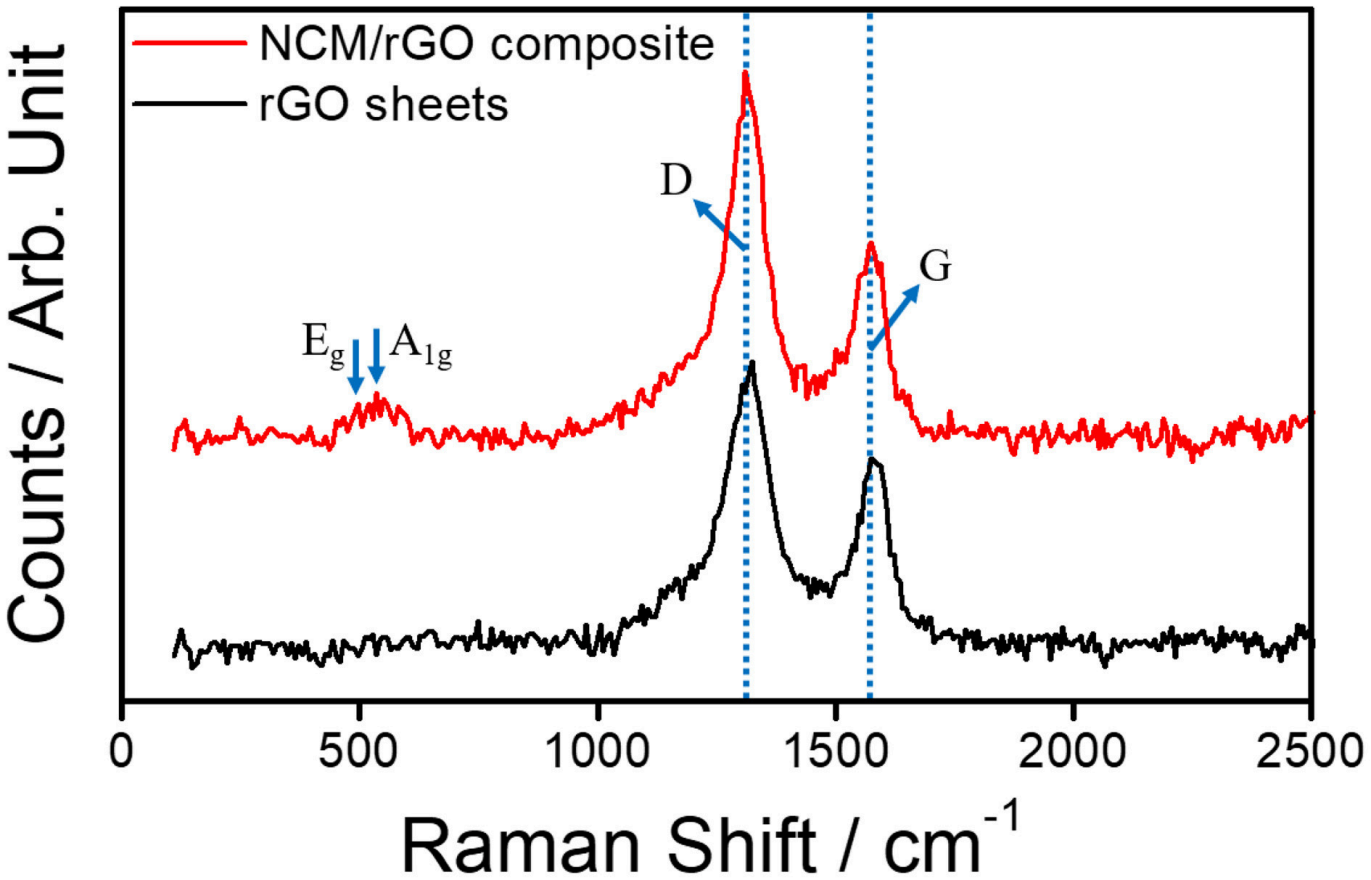
Raman spectra of the pristine rGO sheet and the NCM/rGO composite, which correspond to the specific peak of rGO (D and G) and TM-O (E_g_ and A_1g_), respectively.

Figure 4 shows typical Raman spectra of the rGO sheet and the NCM-rGO composite corresponding to D and G for rGO and E_g_ and A_1g_ for the TM-O bond in the NCM material, respectively. The high intensity of the D-band peak is ascribed to the defects of the graphene layer (*I*_D_/*I*_G_ = 1.7 for the NCM/rGO composite and 1.5 for the rGO sheet), resulting in the NCM/rGO composite being stably synthesized with the decrease in rGO defect. The relative decrease of rGO defect after composite synthesis probably happened due to the bonding effect between edge defect of the rGO sheet and the Triton X-100 surfactant. The two peaks of the NCM/rGO composite near 495 cm^−1^ (E_g_) and 535 cm^−1^ (A_1g_) correspond to specific bands of TM-O arrangements in the layered structure with the R-3m space group (Shim et al., 2017), and the Raman study provides strong evidence that the ultrasonication-assisted self-assembly method could effectively synthesize the NCM/rGO composite without any side reaction.

**Figure 5:**
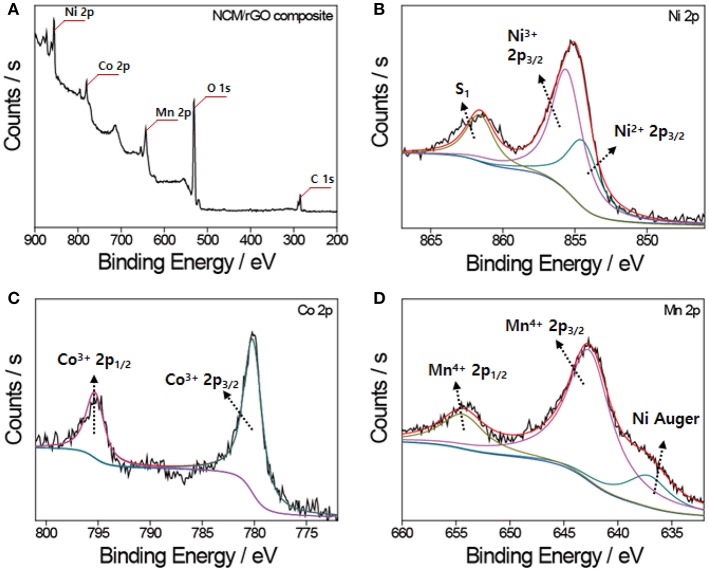
XPS analysis of the NCM/rGO composite. **(A)** The survey XPS spectra of the NCM-rGO composite confirming the existence of expected elements. XPS results of **(B)** Ni 2p spectra, **(C)** Co 2p spectra, and **(D)** Mn 2P spectra. The characteristic peaks of each transition metal element are identified in accordance with our previous research.

The XPS spectra of each transition metal ion presented in Figure 5 very closely match our previously reported work, which confirms no phase transformation and decomposition during the ultrasonication procedure. The XPS survey profile of NCM/rGO presented in Figure 5A also verifies that the high content of the NCM material is homogeneously mixed with the rGO sheet. Figure 5B displays the XPS results of Ni 2p, and the area ratio of the Ni transition metal is decoupled in characteristic oxidation peaks (around 854 and 856 eV), resulting in Ni nearly consisting of 33% Ni^2+^ and 67% Ni^3+^ in the layered structure. The binding energy for each spectrum of Co 2p and Mn 2p (Figures 5C,D) indicates that the Co ion and Mn ion in the sample correspond to the 3+ and 4+ oxidation state, respectively, and this result is perfectly consistent with the theoretical electron valence of the LiNi_0.6_Co_0.2_Mn_0.2_O_2_ material (Tran et al., 2006; Ahn et al., 2014a). From the result of the XRD, it is verified that the cation mixing was not determined, and the low content of Ni^2+^ from XPS analysis also supports the low possibility of Ni/Li cation mixing under raw material preparation by combustion synthesis.

Based on the above physical characterization results, the ultrasonication-assisted self-assembly mechanism of NCM/rGO formation is hypothesized as follows. The surface-activated rGO constructed from rGO with Triton X-100 water suspension suppresses agglomeration of graphene sheets in rGO, resulting in a homogeneously distributed solution. It is noted that the ultrasonication to the rGO sheet is reported to be effective for creating flattened planes of rGO sheets, and this helps to maintain the structure. The highly dispersed flattened rGO sheets then effectively expose a much larger area of graphene surfaces compared to conventional wrinkled rGO sheets that agglomerate much readily, significantly losing their active surface area. This means that the exposure degree of the rGO surface is much larger as well, allowing a higher rate of electrostatic interaction with the NCM material. This prolific interaction between the rGO sheet and the NCM material leads to the generation of a properly distributed composite. In contrast, no ultrasonication under the self-assembly reaction would lead to the formation of separated and aggregated bulk particles either dispersed in the rGO matrix or wrapped by rGO sheets due to the lack of interaction with the exposed surface.

**Figure 6:**
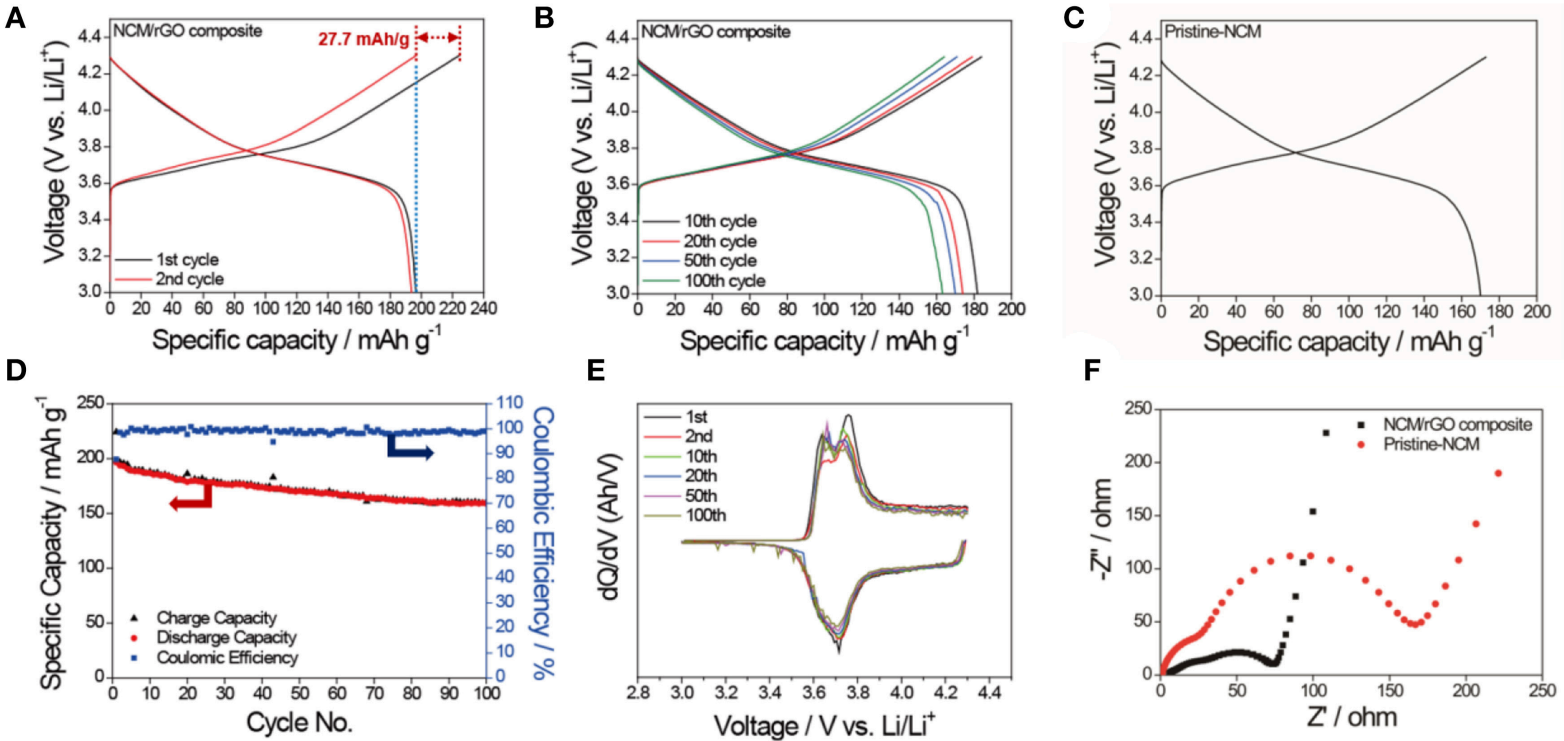
**(A)** The initial charge–discharge profile of the NCM/rGO composite at a current density of 20 mA/g, and **(B)** the stable charge–discharge profiles from the 10th to the 100th cycle. **(C)** The comparison charge–discharge profile of the pristine NCM material, which shows a lower initial capacity than the NCM/rGO composite. **(D)** Cycle durability test and corresponding coulombic efficiency obtained at a current density of 20 mA/g. **(E)** d*Q*/d*V* curves based on the charge–discharge profile from the 1st to the 100th cycle. **(F)** Comparison Nyquist plots obtained from pristine NCM and the NCM/rGO composite.

Having created a unique morphology of NCM nanoparticle-distributed rGO and having elucidated its high impact, the electrochemical performance of the cathode composite for lithium ion battery is demonstrated. Figures 6A,B shows the charge–discharge profile of the NCM/rGO composite obtained by electrochemical evaluations of the half-cell manufacture at a current density of 20 mA/g. It is noted that the NCM622 material has irreversible capacity during the first cycle, with *ca*. 27.7 mAh/g in our study. The first discharge and second charge capacity showed a similar gravimetric capacity of 196.5 mAh/g (see the blue dotted line of the figure), which means only one cycle contributes to the activation and formation of a passive layer on the surface of the cathode material. The average potential during cycle advances is *ca*. 3.75 V until it goes to the 100th cycle, which means that the structure of NCM/rGO is stably maintained. On the other hand, the comparable charge–discharge capacity of the pristine NCM material is shown in Figure 6C. The initial capacity of pristine NCM is ~170 mAh/g, which is lower than that of the NCM/rGO composite. A cycle life and plot of differential capacity (d*Q*/d*V*) vs. potential (*V*) reproduced from the 1st to the 100th discharge–charge profile is presented in Figures 6D,E. Two pairs of peaks are observed on the d*Q*/d*V* vs. *V* plot at 3.67 and 3.77 V (vs. Li/Li^+^) during charge, and 3.63 and 3.71 V (vs. Li/Li^+^) during discharge, respectively. These peaks correspond to the typical characteristic two-step oxidation and reduction reaction of the NCM622 material. As expected, an outstanding initial discharge capacity of 196.5 mAh/g is observed at a current density of 20 mA/g, and the capacity of 163.4 mAh/g with 83.1% capacity retention even after 100 cycles has been achieved. Due to the formation of a passive layer and activation, the coulombic efficiencies of the initial capacity is only 87.7%; however, 98.1% of the average coulombic efficiency obtained during 100 cycles advanced, and the capacity diminution is 0.33 mAh/g per cycle. Furthermore, the capacity retention of the NCM/rGO composite is 163 mAh/g even after 100 cycles, and this result verifies that the superior capacity retention of the NCM/rGO composite is attributed to the direct connectivity between homogenous rGO sheets and the NCM material. In order to compare overall electrical conductivity, EIS measurement was carried out, and typical Nyquist plots obtained from pristine NCM and the NCM/rGO composite are illustrated in Figure 6F. The observed ac-impedance spectra showed relatively low charge transfer resistance for the NCM/rGO composite, which supports an increase in overall electrical conductivity of rGO, and this result is in accordance with the superior initial capacity of the NCM/rGO composite compared with the pristine NCM material. It is noted that the each EIS plot showed two independent semicircles, and the first and second semicircle correspond to the *R*_f_ (surface film resistance) and *R*_ct_ (charge-transfer resistance), which are 48 and 154 Ω for pristine NCM and 41 and 62 Ω for the NCM/rGO composite, respectively. The result in the first semicircle determined that rGO does not affect the formation of a passivation layer, and it only acts as an electric conducting path site for fast charge transfer during advanced electrochemical reaction.

**Figure 7:**
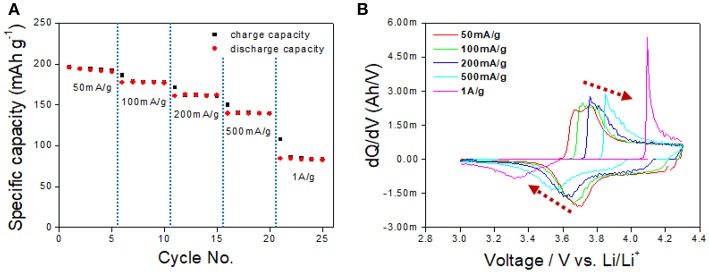
**(A)** The rate capability test at a current density of 50 mA/g to 1 A/g. **(B)** d*Q*/d*V* curve based on the rate capability performance result.

The rate capability and the d*Q*/d*V* vs. *V* plot presented in Figure 7 show initial capacities of 195 and 140 mAh/g obtained at a current density of 50 and 500 mA/g, respectively. The rate capability performance at those current densities demonstrates reliable capacity even at the high-rate cell test, and this is one of the benefits of EV application. In contrast, the capacity at a current density of 1 A/g showed a relatively low capacity of 65 mAh/g, which is caused by the polarization-concentration resistance and IR drop and the low lithium ion diffusivity of the cathode material.

**Figure 8:**
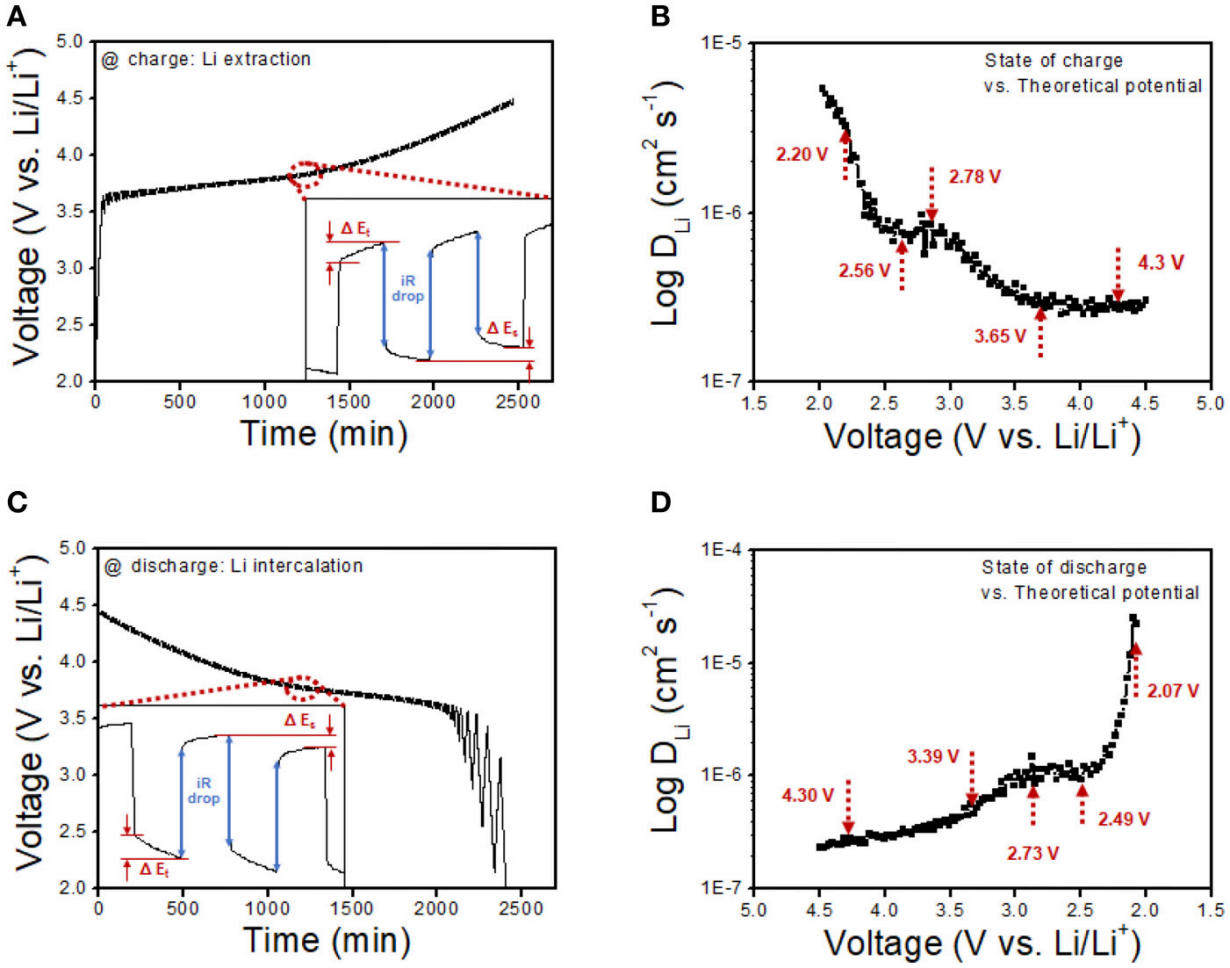
Galvanostatic intermittent titration technique (GITT) curves vs. time at **(A)** the charge procedure and **(C)** the discharge procedure. The duration of the charge and discharge pulses was calculated based on a current density of 20 mA/g. **(B,D)** Lithium diffusivities of the NCM/rGO composite during the charge and discharge procedure as a function of the cell potential. Each inflection point refers to the transformational kinetics in depth of lithium diffusion followed by GITT measurement results.

To further understand the transformation kinetics of lithium ion by calculating practical lithium ion diffusivity during oxidation and reduction reaction under cell operation, GITT measurement was carried out as a function of cell potential. The area and mass loading of the cell are similar with the half-cell test, and the result is presented in Figure 8. It is noticed that the lithium ion diffusivity was calculated by the Weppner–Huggins-derived expression (Hess et al., 2015; Ahn et al., 2016a):

$$\begin{array}{l} D_{Li}=\frac{4{\text{L}}^{2}}{\pi \tau }{\left[\frac{\Delta E_{s}}{\Delta E_{t}}\right]}^{2} \end{array}$$

where *L* and τ refer to the electrode thickness and relaxation time of the current pulse (600 s), respectively, Δ*E*_s_ is the steady-state potential change derived from the current pulse, and Δ*E*_*t*_ is the potential difference during the constant current pulse, eliminating the iR drop. The resultant lithium ion diffusivity as a function of charge and discharge in Figures 8B,D respectively, shows several inflection points, as pointed out by the red arrows. Each inflection point is a determined step of lithium ion diffusion, which is attributed to the dominant region of lithium deintercalation, lithium ion migration as solvated ion in the electrolyte, aggregation on the surface of Li metal, lithium ion plating on the Li metal anode, and the complete lithium reduction on anode during charging step advances. The reversible determined reaction was obtained during discharge with dominant state of lithium stripping from the Li metal anode, lithium ion migration in electrolyte, aggregation on the surface of NCM, and lithium ion diffusion into the bulk particle of NCM. All the GITT results exhibit a similar tendency toward a conventional plot shape for the lithium ion diffusion state. The lithium ion diffusivities of NCM/rGO have been measured at five distinct points as the aforementioned determining steps during charge and discharge procedures, specifying five dominant split reactions as summarized in Table 1 and plotted in Figure 8. First, at the starting point of the charging reaction, the lithium ion begins to diffuse from the bulk NCM particle; then, it starts to migrate toward the organic electrolyte as a solvated ion until reaching 2.20 V. From this step, most of the lithium ions exist inside the bulk particle; thus, the dominant determining step is the migration of the Li ion toward the electrolyte, which is maintained until the concentration of the solvated ion in the electrolyte has increased (2.56 V), resulting in the decrease in lithium ion diffusivity as presented in Figure 8B. Then, lithium ion diffusivity is slightly increased up to 2.78 V, which is attributed to the starting point of aggregation on the surface of the Li anode from the lithium ion migration dominant step, resulting in increased lithium ion diffusivity. The lithium ion starts to plate onto the Li anode from the aggregated solvated ion, and this step is also associated with the decrease in the amount of lithium ion in the electrolyte, which is maintained until 3.65 V. From this step, the lithium ion starts to show low kinetics, which is accompanied by a charge transfer reaction of the lithium ion from a solvated electrolyte molecule to the Li metal anode. Finally, lithium ion diffusivity is stabilized from 3.65 V to the end point of 4.50 V with lowest kinetic reaction in this potential range. The transformation kinetics of the lithium ion during the charge procedure can be divided into four steps, and these individual reaction steps show a similar lithium ion diffusion rate at the discharge procedure. These four distinct steps of transformation kinetic reactions show a similar tendency at the discharge procedure. From the resultant transformation kinetics of the lithium ion during charge–discharge advances, the high lithium ion diffusion could be verified at the cathode electrode part where the lithium ion intercalation–deintercalation procedure was carried out on the surface of the material. It should be noted that lithium ion intercalation–deintercalation is accompanied by charge transfer at the solid–electrolyte interface; hence, the highly electrically conductive material determines the relatively rapid charge transfer of the lithium ion. Even the dominant part of this reaction is lithium ion diffusion in the interlayer of the cathode slab, the charge transfer barrier is the key role in the rate determining step of lithium ion mobility. Therefore, the highly electrically conductive rGO-wrapped NCM composite shows relatively high lithium ion diffusivity (*D*_Li_: 5.34 × 10^−6^) compared with the plating process on the Li metal anode (*D*_Li_: 2.67 × 10^−7^) in this work and our previous study on pristine NCM622 material (24) (*D*_Li_: 4.03 × 10^−14^). As a result, it is explained that the dominant kinetic reaction in each step denotes lithium ion diffusivity, resulting in a correlation with the high electric conductivity of the active material.

**Table 1:** Lithium ion diffusivity of the NCM/rGO composite obtained at five distinct potentials during charge–discharge advances.

**State**	**Electrochemical state at charge**					**Electrochemical state at discharge**				
Potential	2.02 V	2.56 V	2.78 V	3.65 V	4.30 V	4.30 V	3.39 V	3.03 V	2.49 V	2.07 V
*D*_Li_ (cm^2^/s)	5.34 × 10^−6^	6.77 × 10^−7^	9.74 × 10^−7^	2.86 × 10^−7^	2.67 × 10^−7^	2.81 × 10^−7^	5.2 × 10^−7^	9.79 × 10^−7^	9.22 × 10^−7^	2.2 × 10^−6^

## Conclusions

In summary, a homogeneous and uniformly self-assembled rGO-wrapped NCM622 composite for lithium ion batteries has been successfully synthesized by using an ultrasonication-assisted self-assembly method under a Triton X-100 surfactant environment, and the electrochemical properties of cell performance with lithium ion transformation kinetics on the cathode and anode side has been verified. Based on XRD results, ultrasonication indicates a homogeneous mixture of the composite, and the high electrical conductivity of the composite supports this result. The morphology and microstructure of the composite indicated that ultrasonication could effectively pulverize the composite into uniform primary nanoparticles. The rGO-wrapped NCM622 composite exhibits excellent performance with a high specific capacity of 196.5 mAh/g at the initial discharge capacity and 163 mAh/g after 100 cycles with 83% capacity retention at 20 mA/g current density. Furthermore, the discharge capacity with rate capability test showed 140 and 65 mAh/g at 500 mA/g and 1 A/g current density, respectively. The high-rate capability is attributed to the transformation kinetics of lithium ion accompanied by a charge-transfer reaction, which, as confirmed by GITT measurement, likely to have originated from the highly conductive electron paths formed by the perfectly connected NCM622 material on rGO sheets. Based on the enhanced cycle and high-rate performance, it can be concluded that the NCM/rGO composite is a promising cathode material for rechargeable lithium ion batteries, which is highly applicable to the EV field.

## Author Contributions

All authors listed have made substantial, direct and intellectual contribution to the work, and approved it for publication.

### Conflict of Interest Statement

The authors declare that the research was conducted in the absence of any commercial or financial relationships that could be construed as a potential conflict of interest.
